# The effects of acupuncture on sleep disorders and its underlying mechanism: a literature review of rodent studies

**DOI:** 10.3389/fnins.2023.1243029

**Published:** 2023-08-08

**Authors:** Seri Lee, Seung-Nam Kim

**Affiliations:** College of Korean Medicine, Dongguk University, Goyang, Republic of Korea

**Keywords:** sleep disorder, acupuncture, rodent study, literature review, animal study

## Abstract

Sleep is a set of physiological processes mainly under neurobiological regulation that affect several physiological systems, and sleep disorders are a condition where normal sleep patterns are disturbed. Clinical studies have confirmed the effects of acupuncture on sleep duration and quality. Although many studies have explored the therapeutic effects of acupuncture on sleep disorders, the mechanisms are unclear. We investigated the mechanism of acupuncture efficacy in a rodent model of sleep disorders and evaluated the therapeutic effects of acupuncture treatment. According to our results, sleep disorders are associated with several brain regions and neurotransmitters. Furthermore, this review showed that neurological processes, such as catecholamine and BDNF signaling pathways, can be regulated by acupuncture, which is a crucial aspect of the acupuncture mechanism in sleep disorders.

## 1. Introduction

In most humans, sleep accounts for approximately 20–40% of the day. Sleep is a set of physiological processes under neurobiological regulation that affects several physiological systems (Grandner, 2017). Therefore, sufficient sleep is essential for the health of individuals (Kumar, 2008). Sleep disorders are characterized by disturbances in normal sleep patterns (Karna et al., 2023). The six major categories of sleep disorders are insomnia, sleep-disordered breathing, central hypersomnolence disorders, circadian rhythm sleep–wake disorders, parasomnia, and sleep-related movement disorders (Sateia, 2014). Sleep problems are associated with adverse health outcomes such as obesity, cardiovascular diseases, mental health, and neurodegenerative diseases (Hale et al., 2020).

Sleep disorders, such as insomnia or hypersomnia, are frequently observed in neurodegenerative conditions (Malhotra, 2018) including Alzheimer’s disease (AD) and Parkinson’s disease (PD). In individuals with AD, frequent symptoms of sleep disturbances include reversal of day-night sleep patterns, frequent nighttime awakenings, increased daytime sleep, decreased rapid eye movement sleep, and decreased slow wave sleep (Rose and Lorenz, 2010). Regulation of sleep and wakefulness relies on complex functions of several brain areas and neurotransmitters, many of which have been shown to be affected in patients with PD (Stefani and Hogl, 2020). Accordingly, sleep is a complex and active neural process involving several brain structures, such as the hypothalamus, brain stem, amygdala, thalamus, pineal gland, and basal forebrain (Murillo-Rodriguez et al., 2012). Although diverse methods have been used to treat sleep disorders, they lack efficacy and safety (National Institutes of Health, 2005). However, alternative treatments for sleep maintenance are still being developed.

Acupuncture is a therapy based on the insertion of a needle at a specific point, and clear evidence of its efficacy in Western medicine has been reported (Kaptchuk, 2002). The efficacy of acupuncture in improving sleep duration and quality has been confirmed in clinical studies (Cao et al., 2009). Acupuncture in insomnia treatment improves sleep quality and psychological health (Yin et al., 2017). The therapeutic effects of acupuncture in sleep disorders are closely associated with neurological diseases. In particular, acupuncture in PD affects neurotransmitters and their receptors, resulting in increased dopamine, γ-aminobutyric acid (GABA) inhibition, and decreased glutamate levels (Tamtaji et al., 2019). Moreover, acupuncture improves sleep in PD patients (Aroxa et al., 2017; Li et al., 2022).

Although many studies have explored the therapeutic effects of acupuncture on sleep disorders, the mechanisms underlying these effects on sleep disorders are unclear. We evaluated the therapeutic effects of acupuncture treatment and investigated the mechanism of acupuncture efficacy in a rodent model of sleep disorders.

## 2. Methods

### 2.1. Search strategy

We included studies published in English that investigated the effects of acupuncture on sleep disorders in animal models. The literature was retrieved from EMBASE, MEDLINE, PubMed, and the Research Information Service System from inception until April 2023. The keywords for the search were as follows: “(acupuncture OR electroacupuncture) AND (mice OR mouse OR rat OR rats) AND (sleep disorders).” Studies were included based on the following criteria: subjects (animal models of sleep disorders), interventions (acupuncture), and outcomes (electroencephalograms (EEGs) and mechanism). Studies written in languages other than English, those without acute disease models of sleep disorders, or those not needing acupuncture were excluded. Two authors (Lee and Kim) independently extracted the data. The first author, publication year, type of animal, type of sleep disorder and disease model, type of acupuncture, corresponding parameters, and target outcomes were retrieved to evaluate the therapeutic effect of acupuncture on sleep disorders.

### 2.2. Quality assessment

The risk of bias was assessed using the Systematic Review Center for Laboratory Animal Experimentation’s risk of bias (SYRCLE’s RoB) Tool (Hooijmans et al., 2014). The SYRCLE RoB tool contains 10 entries related to selection bias (random sequence generation, baseline characteristics, and allocation concealment), performance bias (random housing and blinding), detection bias (random outcome assessment and blinding), attrition bias (incomplete outcome data), reporting bias (selective reporting), and other biases (other sources of bias). Each entry was marked as “Low risk of bias,” “High risk of bias” or “Unclear.” Two authors (Lee and Kim) independently evaluated the RoB scores of 17 studies. The Review Manager (RevMan) version 5.4 software (The Cochrane Collaboration, 2020) was used to calculate the risk of bias.

## 3. Results

### 3.1. Study inclusion and quality assessment

Among the 43 initially identified studies, 22 studies were removed due to not being written in English and not including acupuncture for treatment. Following full-text screening, four studies that did not provide animal models of inappropriate sleep disorders were excluded. A final total of 17 studies were included in the present study. Quality assessments of the 17 studies were performed by two individual assessors. All studies were rated as having a low risk of sequence generation bias because they mentioned that the animals were randomly grouped. The studies started with animals of similar weights that were maintained under similar environmental conditions. The baseline values of the two groups were similar. Four studies were evaluated as having a high risk of bias in the “incomplete outcome data” domain: not given total number of rats used in the experiment or no explanation for variation of differed number of rats in each experiment. All the studies included the results of their experiments; however, we were unable to determine whether these statements were adequate for the conclusion. Two studies had a high risk of other biases: one study provided insufficient information on electroacupuncture (EA), and one study did not mention the depth of the acupuncture needle during treatment. None of the studies provided information regarding allocation concealment. Flow diagram and quality assessment results were summarized in Supplementary data (Supplementary Figures S1, S2).

### 3.2. Study characteristics

The characteristics of the included studies are summarized in Table 1. Among the 17 studies, all used rats except for one that used mice. EA was the most frequently used intervention. Acupoints located at different sites in the body appear to have multiple uses. Among them, HT7 had the highest number of acupoints.

**Table 1:** Study characteristics.

Author	Disease	Animal	Intervention	Methods	Acupoint
Bo et al. (2017)	Insomnia	rat	MMWA	15 min, 7 days	Dinghui, Heyi, Xin
A et al. (2019)	Insomnia	rat	MWA	15 min, 7 days	GV20, ST36
Hong et al. (2020)	Insomnia	mouse	MA	1 min, 7 days	DU20, SP6, HT7
Xu et al. (2020)	Insomnia	rat	MMWA	15 min, 7 days	Dinghui, Heyi, Xin
Xie et al. (2021)	Insomnia	rat	EA	2 Hz, 6–7 mA, 15 min	BL 18, ST36
Cao et al. (2022)	Insomnia	rat	EA	2/15 Hz, 1 mA	DU20, SP6, HT7
Yu et al. (2022)	Insomnia	rat	MMWA	15 min, 7 days	Dinghui, Heyi, Xin
Zheng et al. (2017)	Sleep deprivation	rat	MA	40 min	EX-HN 1, HT7, SP6
Li et al. (2018)	Sleep deprivation	rat	EA	2 Hz, 120 s	GV20
Chen et al. (2020)	Sleep deprivation	rat	EA	2/100 Hz, 03 mA	GV20, ST36
Pei et al. (2021)	Sleep deprivation	rat	EA	20 min, 4 days	EX-HN1
Hao et al. (2022)	Sleep deprivation	rat	EA	2 Hz, 1 mA, 15 min/day for 7 days	GV20, GV14
Xi et al. (2023)	Chronic sleep deprivation	rat	EA	2 Hz, 1.5 mA	EX-HN 3, HT7
Yi et al. (2015)	Sleep disruption	rat	EA	10 Hz	GB20
Seo et al. (2021)	Sleep disruption	rat	MA	85 Hz, 1 min	HT7
Seo and Ryu (2022)	Sleep disruption	rat	EA	2 Hz, 20 min	HT7
Li et al. (2011)	Sleep disturbance	rat	EA	2 or 100 Hz	ST36, SP6

EA, electroacupuncture; MA, manual acupuncture; MMWA, Mongolian medical warm acupuncture; N/A, not applicable.

### 3.3. Therapeutic effect and its underlying mechanism

We selected 17 studies and highlighted the mechanisms underlying the effects of acupuncture. The mechanisms of each study are summarized in Table 2. Several neurotransmitters and brain sites have been shown to be duplicated in different studies; however, the upregulation of their protein expression levels vary. Therefore, some acupuncture types and acupoints were also duplicated, especially electroacupuncture, which has been shown to be commonly used for treatment of sleep disorders, such as acupoint HT7.

**Table 2:** Effect of acupuncture on sleep disorders and its underlying mechanisms.

Author	Disease	Site of sample collection	Mechanism	EEGs
Bo et al. (2017)	Insomnia	Hippocampus, hypothalamus, prefrontal cortex	miR-101a, 5-HT, ACh, GABA ▲ PAX8, DA, NE, Glu ▼	N/A
A et al. (2019)	Insomnia	Hypothalamus	Egr 1, Btg2, BDNF ▲	N/A
Hong et al. (2020)	Insomnia	Pineal gland, serum	melatonin ▲ DA, 5-HT, NE ▼	N/A
Xu et al. (2020)	Insomnia	Hypothalamus	PRELP, MAP1B, TMEM41B, NMDA, NSMF ▲/▼	N/A
Xie et al. (2021)	Insomnia	Hypothalamus	DA, CRH, ACTH, CORT, D1R, D2R mRNA ▲	NREM ▲
Cao et al. (2022)	Insomnia	Hippocampus, brain stem	5-HT, Bcl-2 ▲ DA, NE, EPI, Bax, Bad, Caspase-3 ▼	N/A
Yu et al. (2022)	Insomnia	Brain stem, serum	ACh, NE, 5-HT, GABA ▲ cAMP, GAT-1 ▼	N/A
Zheng et al. (2017)	Sleep deprivation	Hippocampus	SYP, BDNF ▲	N/A
Li et al. (2018)	Sleep deprivation	Cerebral cortex	N/A	α wave frequency ▲ β wave frequency ▼
Chen et al. (2020)	Sleep deprivation	Hippocampus	p-synapsin I, p-CaMK II, tyrosine hydroxylase ▲	N/A
Pei et al. (2021)	Sleep deprivation	Hippocampus	SYP, PSD95, BDNF/TrkB/Erk pathway ▲	N/A
Hao et al. (2022)	Sleep deprivation	Hippocampus	Rac1, Cdc42 ▲	N/A
Xi et al. (2023)	Chronic sleep deprivation	Hippocampus	DA ▼	N/A
Yi et al. (2015)	Sleep disruption	Central nucleus of amygdala	opioid receptors ▲	NREM ▲
Seo et al. (2021)	Sleep disruption	MS-VDB	c-Fos ▼	NREM ▲
Seo and Ryu (2022)	Sleep disruption	Medial septum	mBDNF ▲	REM ▲
Li et al. (2011)	Sleep disturbance	N/A	N/A	NREM, REM, total sleep time ▲

EA, electroacupuncture; MA, manual acupuncture; MMWA, Mongolian medical warm acupuncture; N/A, not applicable.

The effect of Mongolian medical warm acupuncture (MMWA) on insomnia is related to the regulation of miR-101a and PAX8. MMWA increased the expression of miR-101a which inhibited PAX8 expression in the hippocampus of a rat model of insomnia The levels of 5-HT, Acetylcholine (ACh), and GABA increased and those of dopamine (DA), norepinephrine (NE), and glutamate (Glu) decreased in the hippocampus, hypothalamus, and prefrontal cortex (Bo et al., 2017). Mongolian warm acupuncture (MWA) in a rat model of insomnia improved sleep and the expression levels of Egr1, Btg2, and brain-derived neurotrophic factor (BDNF) in the hypothalamus increased in the MWA treated group (A et al., 2019). Manual acupuncture (MA) stimulation enhanced the sleep disorder phenotype in a rat model of insomnia and had a substantial effect on the recovery of the gut microbiota. Melatonin levels in the pineal gland increased and DA, 5-HT, and NE levels in the serum decreased in the MA treatment group at the DU20, SP6, and HT7 acupoints (Hong et al., 2020). Improvement in sleep was confirmed with MMWA treatment in a rat model of insomnia. Differential levels in the hypothalamus of four proteins related to nerves, including prolargin (PRELP), microtubule-associated protein 1 B (MAP1B), transmembrane protein 41 B (TMEM41B), NMDA receptor synaptonuclear-signaling, and neuronal migration factor (NSMF), are involved in the MMWA treatment of insomnia (Xu et al., 2020). The effect of electroacupuncture treatment (EA) improving sleep disturbance was evaluated in rats with insomnia. The hypothalamic levels of DA, corticotropin-releasing hormone, adrenocorticotropic hormone (ACTH), and cortisol (CORT) increased in the EA group treated at the BL18 and ST36 acupoints. Moreover, EA treatment increased D1R and D2R mRNA levels (Xie et al., 2021). The DU20, HT7, and SP6 acupoints were used for EA treatment in a rat model of insomnia. The EA treatment group showed higher 5-HT levels and lower DA, NE, and EPI levels in the hippocampus and brainstem than the insomnia group. The expression of Bcl-2 was upregulated, whereas Bax, Bad and Caspase-3 were downregulated. EA affects the TrkB, PI3K/Akt, and cAMP/CREB/BDNF pathways (Cao et al., 2022). The anti-insomnia effect of MMWA has been observed in rats with insomnia as MMWA treatment decreased ACh and NE levels and increased 5-HT and GABA levels in the serum. The gut microbiota improved, and cAMP and GABA transporter 1 (GAT-1) levels in the brain stem were reduced (Yu et al., 2022).

The treatment of sleep-deprived rats with acupuncture affected their learning and memory. The expression levels of the presynaptic marker synaptophysin (SYP) and BDNF in the hippocampus of the acupuncture group treated with HT7, EX-HN1, and SP6 were higher than those in the sleep deprivation (SD) group (Zheng et al., 2017). The effects of EA on memory were assessed using an SD rat model. Compared with the SD group, the EA group treated at the GV20 and ST36 acupoints showed higher p-synapsin I, p-CaMK II, and tyrosine hydroxylase levels in the hippocampus (Chen et al., 2020). Compared with the SD group, more neurons were observed in the CA1 and CA2 regions of the hippocampus in the EA group treated at the EX-HN1 acupoint. The expression levels of SYP and the postsynaptic marker, postsynaptic density (PSD) 95, increased in the EA group compared to those in the SD group. Furthermore, EA activates the BDNF/TrkB/Erk pathway (Pei et al., 2021). Two acupoints, GV20 and GV14, were used for EA treatment in a REM sleep deprivation (REMSD) rat model. EA alleviates damage to the synaptic ultrastructure and upregulates dendrite branching and length in the hippocampus in the REMSD rat model. Structural synaptic plasticity in REMSD model rats was attenuated by EA regulation of miR-132-3p and p250GAP. Moreover, EA elevated the expression of Rac1 and Cdc42 in the hippocampus. EA treatment increases the expression of Rac1 and Cdc42, which are connected to miR-132 (Hao et al., 2022). The EA group treated with EX-HN3 and HT7 in a rat model of chronic sleep deprivation reduced DA expression via the VTA-NAc DA pathway (Xi et al., 2023).

EA treatment suppresses epilepsy and improves sleep disruption at the GB20 acupoint in rats with epilepsy. NREM sleep reduction is blocked by EA treatment, and the therapeutic effect is mediated by opioid receptors in the central nucleus of the amygdala (CeA) (Yi et al., 2015). The effect of mechanical acupuncture instrument (MAI) treatment was induced by the HT7 acupoint in a rat model of sleep disruption. MAI stimulation reduced c-Fos expression in arousal regions, especially the medial septum/vertical limb of the diagonal band of Broca (MS-VDB) (Seo et al., 2021). EA treatment of HT7 cells alleviates endoplasmic reticulum (ER) stress in the medial septum of rats with sleep disruption. HT7 stimulation increased the expression of mBDNF and regulated ER stress via pTrkB in mBDNF (Seo and Ryu, 2022).

## 4. Discussion

**Figure 1:**
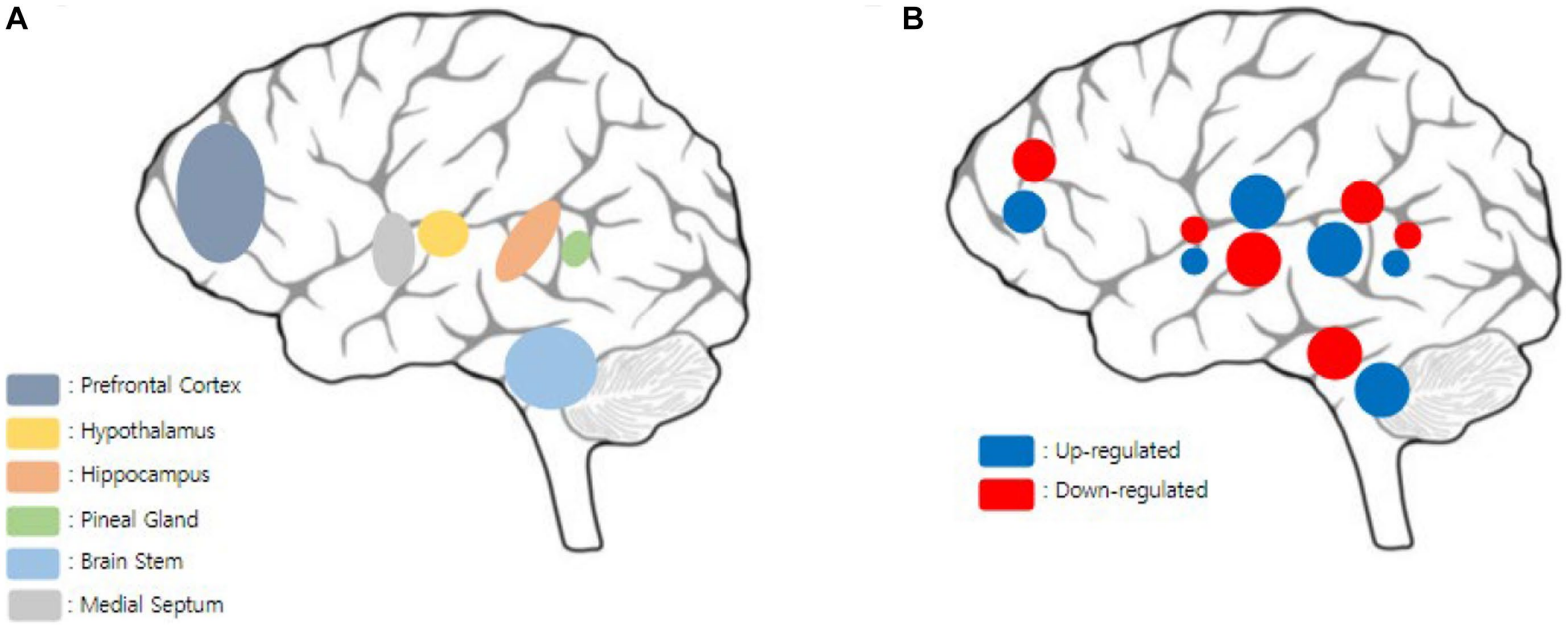
Visualization of mechanisms via acupuncture stimulation in multiple brain regions. **(A)** Multiple brain regions that acupuncture mechanisms are involved in this report. Various brain regions were detected such as prefrontal cortex, hypothalamus, hippocampus, pineal gland, brain stem, and medial septum. **(B)** Expression levels such as neurotransmitters, proteins, or genes which are involved in multiple brain regions according to this report. Each color pretends the up/down regulations of expression levels, and the size of the circle pretends the number of catecholamine types analyzed in each brain regions. In the prefrontal cortex, miR-101a, 5-HT, Ach, GABA were up regulated, and PAX8, DA, NE, and Glu down regulated. In, medial septum, mBDNF were up regulated and c-Fos were down regulated. In the hypothalamus, miR-101a, 5-HT, Ach, GABA, Egr 1, Btg 2, BDNF, DA, CRH, ACTH, CORT, D1R and D2R mRNA, were up regulated, and PAX8, DA, NE, Glu, were down regulated. PRELP, MAP1B, TMEM41B, NMDA, NSMF were shown have differential expression levels. In the hippocampus, miR-101a, 5-HT, Ach, GABA, Bcl-2, SYP, BDNF, p-synapsin I, p-CaMK II, tyrosine hydroxylase, PSD95, BDNF/TrkB/Erk pathway, Rac1, Cdc42 were up regulated, and PAX8, DA, NE, Glu, EPI, Bax, Bad, Caspase-3 were down regulated. In the pineal gland, melatonin was up regulated and DA, 5-HT, NE were down regulated. In the brain stem, miR-101a, 5-HT, Ach, GABA, Bcl-2, NE, were up regulated, and PAX8, DA, NE, Glu, EPI, Bax, Bad, Caspase-3, cAMP, GAT-1 were down regulated.

This study explored the beneficial effects of acupuncture in animal models of sleep disorders and the underlying mechanisms. According to the results of our study, sleep disorders are associated with several brain regions and neurotransmitter levels. Various brain regions and expression levels that represent the acupuncture mechanism described in the main results are shown in Figure 1. Sleep is closely intertwined with physiological processes, typically in the brain (Lin et al., 2014). Previous studies have presented the neuroprotective activity and neurotransmitter regulation of acupuncture therapy (Su et al., 2020). BDNF is a protein that is extensively distributed in the cartilage tissue, bone, endocrine system, and central nervous system (CNS) (Greenberg et al., 2009), and is widely involved in neural plasticity (Park and Poo, 2013; Colucci-D'Amato et al., 2020). Studies have also demonstrated that acupuncture induces advantages in the central nervous system (CNS) through BDNF activation and signaling pathways (Lin et al., 2014). Two studies confirmed the effect of acupuncture on BDNF expression levels (Zheng et al., 2017; A et al., 2019). Moreover, various downstream pathways, such as the TrKB, PI3K/Akt, cAMP/CREB/BDNF (Cao et al., 2022), and BDNF/TrkB/Erk pathways (Pei et al., 2021), were found to be influenced by acupuncture treatment. BDNF appears to be essential for mediating neuroprotective effects and may play a role in neuronal plasticity (Colucci-D'Amato et al., 2020). Therefore, the effects of acupuncture on the regulation of BDNF appear to be important for its role in treating sleep dysfunction.

Changes in neurotransmitter levels such as DA, 5-HT, NE, Glu, and GABA in various brain regions lead to neuropsychiatric, autonomic nervous system, and sleep disorders (Zhao et al., 2021). Because neurological processes are involved in sleep–wake regulation (Scammell, 2015), the interaction of diverse neurotransmitter systems is widely related to sleep dysfunction (Moszczynski and Murray, 2012). Acupuncture therapy, which has beneficial effects on neuroprotection, has been shown to improve serum and hippocampal 5-HT and DA levels in depression model rats (Li et al., 2021). Interestingly, several studies have shown increased levels of 5-HT expression in different sites, such as the serum (Yu et al., 2022), hypothalamus (Bo et al., 2017; Xie et al., 2021), and hippocampus (Bo et al., 2017; Cao et al., 2022) following acupuncture treatment. Among these studies, one showed an improvement in both 5-HT and DA expression levels (Xie et al., 2021). Moreover, according to previous reports, dopaminergic neurons are typically found in the midbrain, particularly in the VTA (Juarez Olguin et al., 2016). In this review, the VTA-NAc DA pathway was demonstrated to be regulated through acupuncture stimulation in one study (Xi et al., 2023). Two studies have reported decreased levels of DA and increased levels of 5-HT (Bo et al., 2017; Cao et al., 2022). In contrast, one study reported downregulated levels of DA and 5-HT (Hong et al., 2020). This review demonstrates that catecholamines are regulated by acupuncture in animal models of sleep disorders.

In this review, various acupoints were used for acupuncture treatment. Among these acupuncture points, HT7 was the most frequently used. According to recent studies, HT7 has been used for neuropsychological disorders such as amnesia, epilepsy, and insomnia (Wattanathorn and Sutalangka, 2014). In this study, stimulation of HT7 mediated memory improvements and ACh and DA levels were enhanced by HT7. However, the acupoints ST36 (Tao et al., 2016), GV20 (Wang et al., 2014), and SP6 (Wu et al., 2015) have also been used to mitigate sleep dysfunction. Interestingly, these acupoints have been known to have neuroprotective effects on various neurological disease animal models. Sleep patterns and insomnia symptoms seem to correlate with brain injury (Korostovtseva, 2021) and an imbalance in neurotransmitters (Levenson et al., 2015; Holst and Landolt, 2018). Therefore, this difference in mechanism suggests a distinct and specific effect of acupuncture on the treated acupoints. Along with the effects of acupuncture therapy, multiple acupoints and their potential roles in various mechanisms may be crucial for establishing a strategy for treating sleep disorders.

Understanding sleep regulation may encourage the management of neurocognitive disorders (Miller, 2015) and chronic diseases (Reis et al., 2018). Although this review had a small sample size, we attempted to explore the potential effect of acupuncture on sleep disorders in neuropsychology and to understand its mechanism in animal models. Accordingly, the present study is meaningful and important in exploring the correlation between acupuncture and neuropsychological disorders in animal models. However, multiple interconnections between acupuncture, sleep problems, and neuroscience still need to be described. We hope that this report will provide a basis for exploring the mechanisms of action of acupuncture in neuropsychological disorders.

## Author contributions

SL searched the database and extracted the data. S-NK designed and supervised the study. SL and S-NK analyzed the data and wrote the paper. All authors contributed to the article and approved the submitted version.

## Funding

This work was supported by the National Research Foundation of Korea funded by the Korean government (MSIT) (NRF-2020R1C1C1004107) and from the Ministry of Health and Welfare through the Korea Health Industry Development Institute (KHIDI) (grant no. HF21C0018).

## Conflict of interest

The authors declare that the research was conducted in the absence of any commercial or financial relationships that could be construed as a potential conflict of interest.

## Publisher’s note

All claims expressed in this article are solely those of the authors and do not necessarily represent those of their affiliated organizations, or those of the publisher, the editors and the reviewers. Any product that may be evaluated in this article, or claim that may be made by its manufacturer, is not guaranteed or endorsed by the publisher.

## References

[ref1] A G. Li X. Su B. Lian H. Bao M. Liang Y.. (2019). Effect of Mongolian warm acupuncture on the gene expression profile of rats with insomnia. Acupunct. Med. 37, 301–311. doi: 10.1136/acupmed-2016-011243, PMID: 31225736

[ref2] Aroxa F. H. Gondim I. T. Santos E. L. Coriolano M. D. Asano A. G. Asano N. M. (2017). Acupuncture as adjuvant therapy for sleep disorders in Parkinson's disease. J. Acupunct. Meridian Stud. 10, 33–38. doi: 10.1016/j.jams.2016.12.007, PMID: 28254099

[ref3] Bo A. Si L. Wang Y. Bao L. Yuan H. (2017). Mechanism of Mongolian medical warm acupuncture in treating insomnia by regulating miR-101a in rats with insomnia. Exp. Ther. Med. 14, 289–297. doi: 10.3892/etm.2017.4452, PMID: 28672928 PMC5488598

[ref4] Cao H. Pan X. Li H. Liu J. (2009). Acupuncture for treatment of insomnia: a systematic review of randomized controlled trials. J. Altern. Complement. Med. 15, 1171–1186. doi: 10.1089/acm.2009.0041, PMID: 19922248 PMC3156618

[ref5] Cao F. Xu Y. Zhang M. Li X. Chen Y. Zhi M.. (2022). Baihui (DU20), Shenmen (HT7) and Sanyinjiao (SP6) target the cAMP/CREB/BDNF and PI3K/Akt pathways to reduce central nervous system apoptosis in rats with insomnia. Heliyon. 8:e12574. doi: 10.1016/j.heliyon.2022.e12574, PMID: 36636219 PMC9830165

[ref6] Chen D. Zhang Y. Wang C. Wang X. Shi J. Zhang J.. (2020). Modulation of hippocampal dopamine and synapse-related proteins by electroacupuncture improves memory deficit caused by sleep deprivation. Acupunct. Med. 38, 343–351. doi: 10.1177/0964528420902147, PMID: 32370535

[ref7] Colucci-D'Amato L. Speranza L. Neurotrophic V. F. Factor B. D. N. F. (2020). Physiological functions and therapeutic potential in depression, neurodegeneration and brain Cancer. Int. J. Mol. Sci. 21, 1–29. doi: 10.3390/ijms21207777, PMID: 33096634 PMC7589016

[ref8] Grandner M. A. (2017). Sleep, health, and society. Sleep Med. Clin. 12, 1–22. doi: 10.1016/j.jsmc.2016.10.012, PMID: 28159089 PMC6203594

[ref9] Greenberg M. E. Xu B. Lu B. Hempstead B. L. (2009). New insights in the biology of BDNF synthesis and release: implications in CNS function. J. Neurosci. 29, 12764–12767. doi: 10.1523/JNEUROSCI.3566-09.2009, PMID: 19828787 PMC3091387

[ref10] Hale L. Troxel W. Buysse D. J. (2020). Sleep health: an opportunity for public health to address health equity. Annu. Rev. Public Health 41, 81–99. doi: 10.1146/annurev-publhealth-040119-094412, PMID: 31900098 PMC7944938

[ref11] Hao L. Wu Y. Xie J. Chen X. (2022). Electroacupuncture enhances cognitive deficits in a rat model of rapid eye movement sleep deprivation via targeting MiR-132. Evid. Based Complement. Alternat. Med. 2022, 7044208–7044214. doi: 10.1155/2022/7044208, PMID: 36159559 PMC9507748

[ref12] Holst S. C. Landolt H. P. (2018). Sleep-Wake Neurochemistry. Sleep Med. Clin. 13, 137–146. doi: 10.1016/j.jsmc.2018.03.002, PMID: 29759265

[ref13] Hong J. Chen J. Kan J. Liu M. Yang D. (2020). Effects of acupuncture treatment in reducing sleep disorder and gut microbiota alterations in PCPA-induced insomnia mice. Evid. Based Complement. Alternat. Med. 2020, 3626120–3626114. doi: 10.1155/2020/3626120, PMID: 33178314 PMC7647758

[ref14] Hooijmans C. R. Rovers M. M. de Vries R. B. Leenaars M. Ritskes-Hoitinga M. Langendam M. W. (2014). SYRCLE's risk of bias tool for animal studies. BMC Med. Res. Methodol. 14:43. doi: 10.1186/1471-2288-14-43, PMID: 24667063 PMC4230647

[ref15] Juarez Olguin H. Calderon Guzman D. Hernandez Garcia E. Barragan M. G. (2016). The role of dopamine and its dysfunction as a consequence of oxidative stress. Oxidative Med. Cell. Longev. 2016, 9730467–9730413. doi: 10.1155/2016/9730467, PMID: 26770661 PMC4684895

[ref16] Kaptchuk T. J. (2002). Acupuncture: theory, efficacy, and practice. Ann. Intern. Med. 136, 374–383. doi: 10.7326/0003-4819-136-5-200203050-00010 11874310

[ref17] Karna B Sankari A Tatikonda G. (2023). Sleep Disorder. StatPearls. Treasure Island, FL. 32809555

[ref18] Korostovtseva L. (2021). Ischemic stroke and sleep: the linking genetic factors. Cardiol. Ther. 10, 349–375. doi: 10.1007/s40119-021-00231-9, PMID: 34191267 PMC8555086

[ref19] Kumar V. M. (2008). Sleep and sleep disorders. Indian J. Chest Dis. Allied Sci. 50, 129–135. 18610697

[ref20] Levenson J. C. Kay D. B. Buysse D. J. (2015). The pathophysiology of insomnia. Chest 147, 1179–1192. doi: 10.1378/chest.14-1617, PMID: 25846534 PMC4388122

[ref21] Li P. Huang W. Yan Y. N. Cheng W. Liu S. Huang Y.. (2021). Acupuncture can play an antidepressant role by regulating the intestinal microbes and neurotransmitters in a rat model of depression. Med. Sci. Monit. 27:e929027. doi: 10.12659/MSM.929027, PMID: 34039946 PMC8168287

[ref22] Li L. Jin X. Cong W. Du T. Zhang W. (2022). Acupuncture in the treatment of Parkinson's disease with sleep disorders and dose response. Biomed. Res. Int. 2022:7403627. doi: 10.1155/2022/7403627, PMID: 35252455 PMC8890872

[ref23] Li J. Ran X. Cui C. Xiang C. Zhang A. Shen F. (2018). Instant sedative effect of acupuncture at GV20 on the frequency of electroencephalogram alpha and beta waves in a model of sleep deprivation. Exp. Ther. Med. 15, 5353–5358. doi: 10.3892/etm.2018.6123, PMID: 29896222 PMC5994783

[ref24] Li Y. J. Zhong F. Yu P. Han J. S. Cui C. L. Wu L. Z. (2011). Electroacupuncture treatment normalized sleep disturbance in morphine withdrawal rats. Evid. Based Complement. Alternat. Med. 2011:361054. doi: 10.1093/ecam/nep133, PMID: 19734257 PMC3137251

[ref25] Lin D. De La Pena I. Lin L. Zhou S. F. Borlongan C. V. Cao C. (2014). The neuroprotective role of acupuncture and activation of the BDNF signaling pathway. Int. J. Mol. Sci. 15, 3234–3252. doi: 10.3390/ijms15023234, PMID: 24566146 PMC3958908

[ref26] Malhotra R. K. (2018). Neurodegenerative Disorders and Sleep. Sleep Med. Clin. 13, 63–70. doi: 10.1016/j.jsmc.2017.09.006 29412984

[ref27] Miller M. A. (2015). The role of sleep and sleep disorders in the development, diagnosis, and management of neurocognitive disorders. Front. Neurol. 6:224. doi: 10.3389/fneur.2015.00224, PMID: 26557104 PMC4615953

[ref28] Moszczynski A. Murray B. J. (2012). Neurobiological aspects of sleep physiology. Neurol. Clin. 30, 963–985. doi: 10.1016/j.ncl.2012.08.001 23099125

[ref29] Murillo-Rodriguez E. Arias-Carrion O. Zavala-Garcia A. Sarro-Ramirez A. Huitron-Resendiz S. Arankowsky-Sandoval G. (2012). Basic sleep mechanisms: an integrative review. Cent. Nerv. Syst. Agents Med. Chem. 12, 38–54. doi: 10.2174/187152412800229107 22524274

[ref30] National Institutes of Health (2005). National Institutes of Health state of the science conference statement on manifestations and Management of Chronic Insomnia in adults, June 13–15, 2005. Sleep 28, 1049–1057. doi: 10.1093/sleep/28.9.1049, PMID: 16268373

[ref31] Park H. Poo M. M. (2013). Neurotrophin regulation of neural circuit development and function. Nat. Rev. Neurosci. 14, 7–23. doi: 10.1038/nrn3379 23254191

[ref32] Pei W. Meng F. Deng Q. Zhang B. Gu Y. Jiao B.. (2021). Electroacupuncture promotes the survival and synaptic plasticity of hippocampal neurons and improvement of sleep deprivation-induced spatial memory impairment. CNS Neurosci. Ther. 27, 1472–1482. doi: 10.1111/cns.13722, PMID: 34623740 PMC8611786

[ref33] Reis C. Dias S. Rodrigues A. M. Sousa R. D. Gregorio M. J. Branco J.. (2018). Sleep duration, lifestyles and chronic diseases: a cross-sectional population-based study. Sleep Sci 11, 217–230. doi: 10.5935/1984-0063.20180036, PMID: 30746039 PMC6361301

[ref34] Rose K. M. Lorenz R. (2010). Sleep disturbances in dementia. J. Gerontol. Nurs. 36, 9–14. doi: 10.3928/00989134-20100330-05, PMID: 20438013 PMC3062259

[ref35] Sateia M. J. (2014). International classification of sleep disorders-third edition: highlights and modifications. Chest 146, 1387–1394. doi: 10.1378/chest.14-0970 25367475

[ref36] Scammell T. E. (2015). Overview of sleep: the neurologic processes of the sleep-wake cycle. J. Clin. Psychiatry 76:e13. doi: 10.4088/JCP.14046tx1c 26035194

[ref37] Seo S. Y. Moon J. Y. Kang S. Y. Kwon O. S. Bang S. K. Choi K. H.. (2021). Acupuncture stimulation at HT7 as a non-pharmacological therapy for sleep disorder caused by caffeine administration in rats. Acupunct. Med. 39, 691–699. doi: 10.1177/09645284211011489, PMID: 34056932

[ref38] Seo S. Y. Ryu Y. (2022). Electroacupuncture stimulation of HT7 alleviates sleep disruption following acute caffeine exposure by regulating BDNF-mediated endoplasmic reticulum stress in the rat medial septum. Biomed. Pharmacother. 155:113724. doi: 10.1016/j.biopha.2022.113724, PMID: 36156370

[ref39] Stefani A. Hogl B. (2020). Sleep in Parkinson's disease. Neuropsychopharmacology 45, 121–128. doi: 10.1038/s41386-019-0448-y, PMID: 31234200 PMC6879568

[ref40] Su X. T. Wang L. Ma S. M. Cao Y. Yang N. N. Lin L. L.. (2020). Mechanisms of acupuncture in the regulation of oxidative stress in treating ischemic stroke. Oxidative Med. Cell. Longev. 2020, 7875396–7875315. doi: 10.1155/2020/7875396, PMID: 33178387 PMC7644298

[ref41] Tamtaji O. R. Naderi Taheri M. Notghi F. Alipoor R. Bouzari R. Asemi Z. (2019). The effects of acupuncture and electroacupuncture on Parkinson's disease: current status and future perspectives for molecular mechanisms. J. Cell. Biochem. 120, 12156–12166. doi: 10.1002/jcb.28654, PMID: 30938859

[ref42] Tao J. Zheng Y. Liu W. Yang S. Huang J. Xue X.. (2016). Electro-acupuncture at LI11 and ST36 acupoints exerts neuroprotective effects via reactive astrocyte proliferation after ischemia and reperfusion injury in rats. Brain Res. Bull. 120, 14–24. doi: 10.1016/j.brainresbull.2015.10.011, PMID: 26524137

[ref43] Wang W. W. Xie C. L. Lu L. Zheng G. Q. (2014). A systematic review and meta-analysis of Baihui (GV20)-based scalp acupuncture in experimental ischemic stroke. Sci. Rep. 4:3981. doi: 10.1038/srep03981, PMID: 24496233 PMC5379241

[ref44] Wattanathorn J. Sutalangka C. (2014). Laser acupuncture at HT7 Acupoint improves cognitive deficit, neuronal loss, oxidative stress, and functions of cholinergic and dopaminergic Systems in Animal Model of Parkinson's disease. Evid. Based Complement. Alternat. Med. 2014:937601. doi: 10.1155/2014/937601, PMID: 25161693 PMC4138813

[ref45] Wu M. F. Zhang S. Q. Liu J. B. Li Y. Zhu Q. S. Gu R. (2015). Neuroprotective effects of electroacupuncture on early- and late-stage spinal cord injury. Neural Regen. Res. 10, 1628–1634. doi: 10.4103/1673-5374.167762, PMID: 26692861 PMC4660757

[ref46] Xi H. Wu W. Qin S. Wang X. Liu C. (2023). Effects of electroacupuncture on the ventral tegmental area- nucleus accumbens dopamine pathway in rats with chronic sleep deprivation. Acupunct. Med.:9645284221146197. doi: 10.1177/09645284221146197, PMID: 36655631

[ref47] Xie C. Wang J. Zhao N. Yang W. Gao X. Liu Z.. (2021). Effects of Electroacupuncture on sleep via the dopamine system of the HPA axis in rats after cage change. Evid. Based Complement. Alternat. Med. 2021, 5527060–5527025. doi: 10.1155/2021/5527060, PMID: 34306138 PMC8270700

[ref48] Xu Y. Li X. Man D. Su X. A G. (2020). iTRAQ-based proteomics analysis on insomnia rats treated with Mongolian medical warm acupuncture. Biosci. Rep. 40, 1–13. doi: 10.1042/BSR20191517, PMID: 32249904 PMC7953503

[ref49] Yi P. L. Lu C. Y. Jou S. B. Chang F. C. (2015). Low-frequency electroacupuncture suppresses focal epilepsy and improves epilepsy-induced sleep disruptions. J. Biomed. Sci. 22:49. doi: 10.1186/s12929-015-0145-z, PMID: 26150021 PMC4491875

[ref50] Yin X. Gou M. Xu J. Dong B. Yin P. Masquelin F.. (2017). Efficacy and safety of acupuncture treatment on primary insomnia: a randomized controlled trial. Sleep Med. 37, 193–200. doi: 10.1016/j.sleep.2017.02.012 28899535

[ref51] Yu H. Yu H. Si L. Meng H. Chen W. Wang Z.. (2022). Influence of warm acupuncture on gut microbiota and metabolites in rats with insomnia induced by PCPA. PLoS One 17:e0267843. doi: 10.1371/journal.pone.0267843, PMID: 35482778 PMC9049555

[ref52] Zhao Y. Zhang Z. Qin S. Fan W. Li W. Liu J.. (2021). Acupuncture for Parkinson's disease: efficacy evaluation and mechanisms in the dopaminergic neural circuit. Neural Plast. 2021:9926445. doi: 10.1155/2021/9926445, PMID: 34221005 PMC8221898

[ref53] Zheng P. Xu X. Zhao H. Lv T. Song B. Wang F. (2017). Tranquilizing and allaying excitement needling method affects BDNF and SYP expression in Hippocampus. Evid. Based Complement. Alternat. Med. 2017:8215949. doi: 10.1155/2017/8215949, PMID: 28761498 PMC5518541

