# Supplementary material for: The effects of acupuncture on sleep disorders and its underlying mechanism: a literature review of rodent studies
Source: Front Neurosci. 2023 Aug 8;17:1243029. doi: 10.3389/fnins.2023.1243029 (PMC10442542; doi:10.3389/fnins.2023.1243029)

The effects of acupuncture on sleep disorders and its underlying mechanism: A literature review of rodent studies

Seri Lee^1^ and Seung-Nam Kim^1*^

^1^College of Korean Medicine, Dongguk University, Goyang 10326, Republic of Korea

*** Correspondence:**Seung-Nam Kim
[snkim@dongguk.edu](mailto:snkim@dongguk.edu)

This is the Supplementary data file of the original manuscript.

**Supplementary Figure 1. Flow diagram of the process for the study inclusion.**


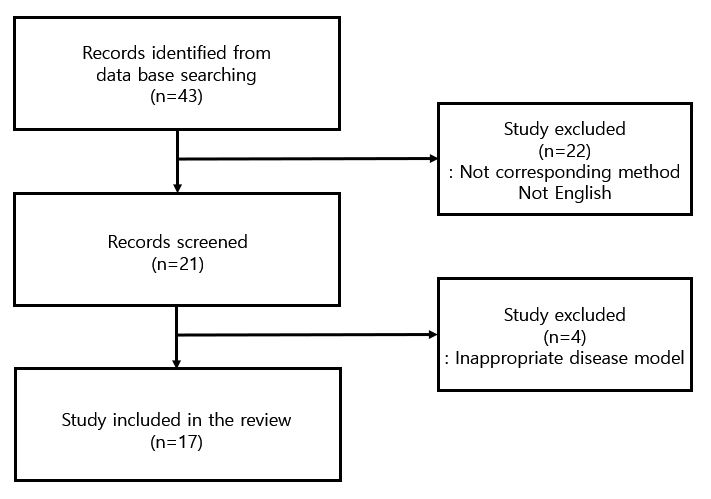


**Supplementary Figure 2. Quality assessment.**


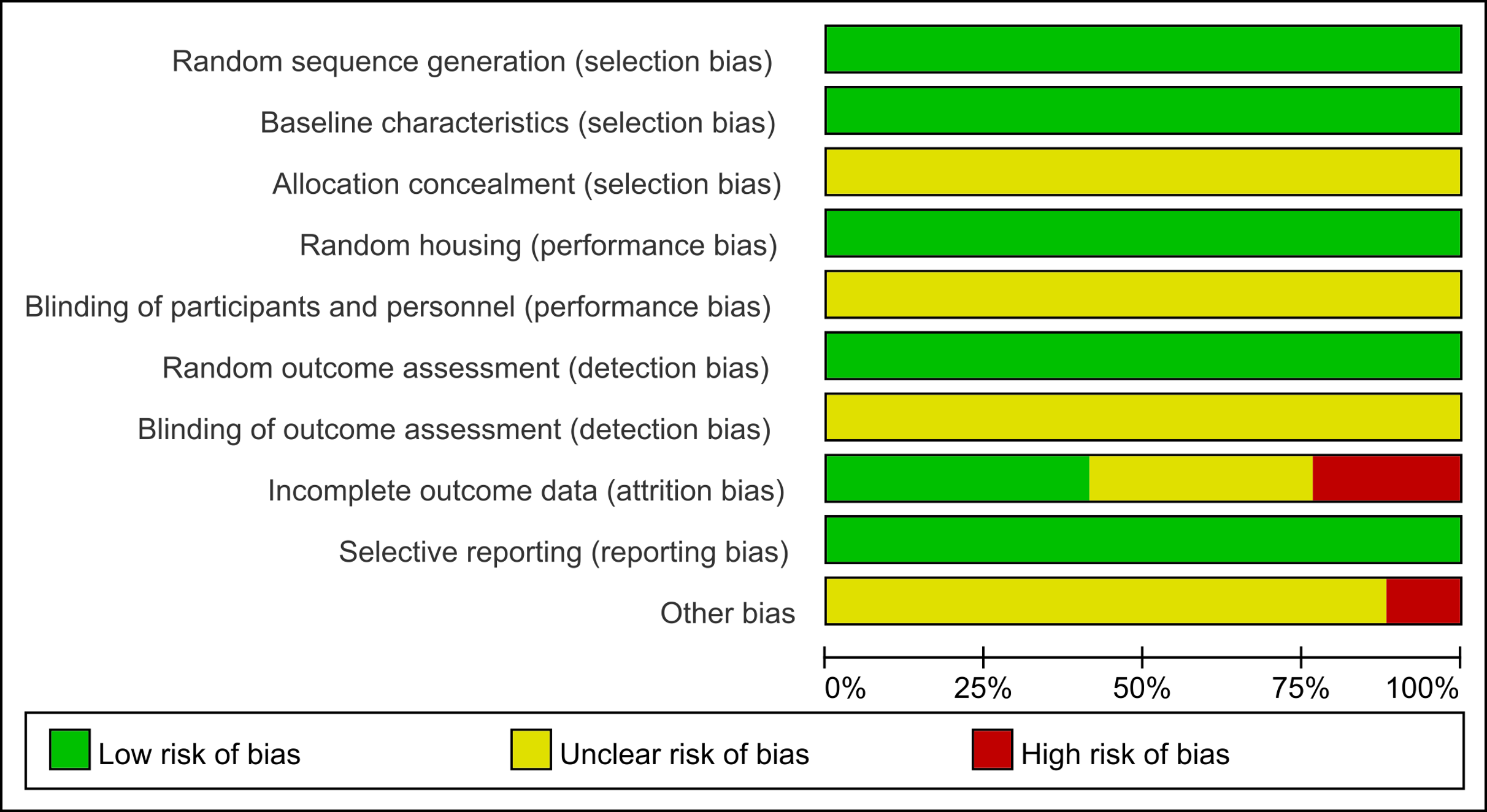


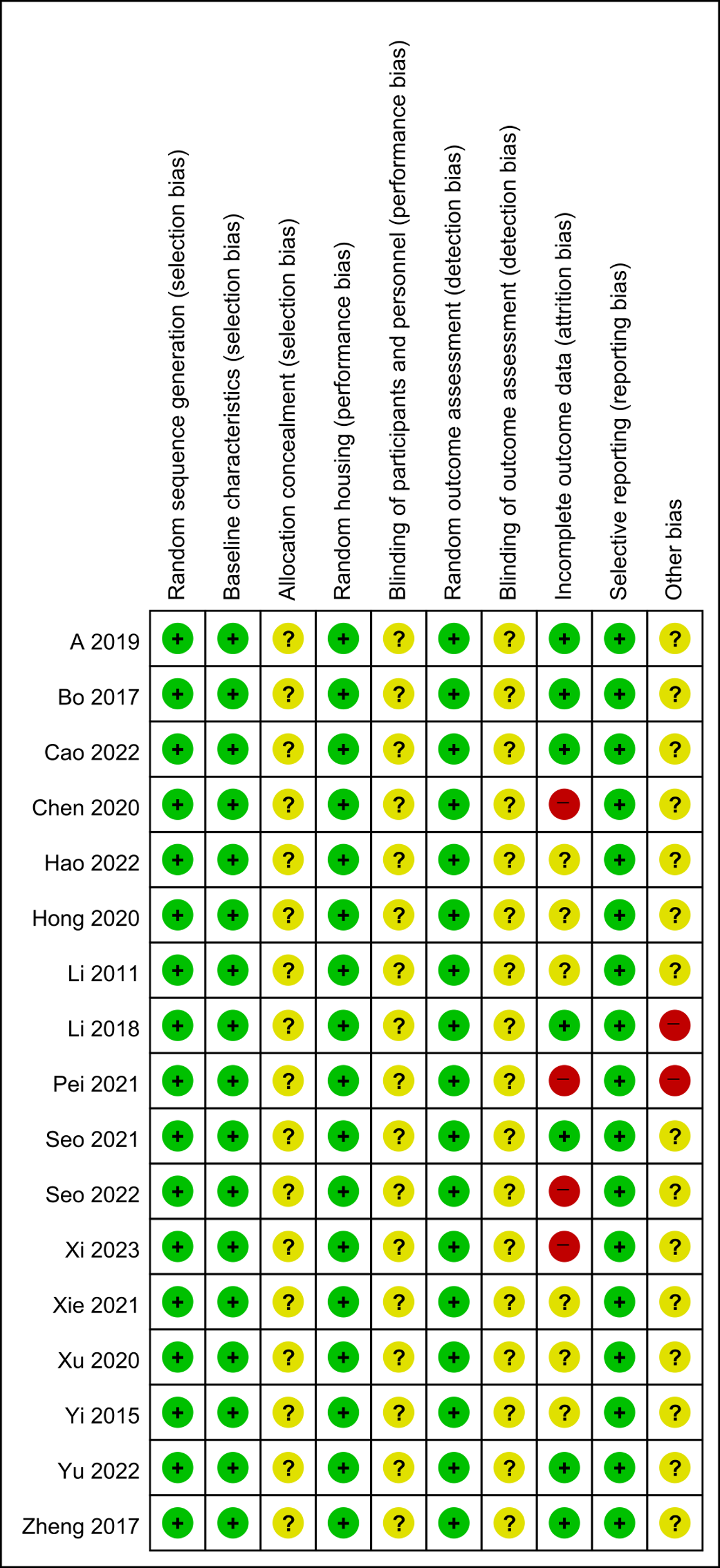

Supplement: Supplementary file 1 [file Data_Sheet_1.docx]
